# Modeling DNA affinity landscape through two-round support vector regression with weighted degree kernels

**DOI:** 10.1186/1752-0509-8-S5-S5

**Published:** 2014-12-12

**Authors:** Xiaolei Wang, Hiroyuki Kuwahara, Xin Gao

**Affiliations:** 1 Computer, Electrical and Mathematical Sciences and Engineering Division, King Abdullah University of Science and Technology (KAUST), 23955 Thuwal, Kingdom of Saudi Arabia; 2 Computational Bioscience Research Center, King Abdullah University of Science and Technology (KAUST), 23955 Thuwal, Kingdom of Saudi Arabia

**Keywords:** binding affinity, protein-DNA interaction, support vector regression, weighted degree kernel

## Abstract

**Background:**

A quantitative understanding of interactions between transcription factors (TFs) and their DNA binding sites is key to the rational design of gene regulatory networks. Recent advances in high-throughput technologies have enabled high-resolution measurements of protein-DNA binding affinity. Importantly, such experiments revealed the complex nature of TF-DNA interactions, whereby the effects of nucleotide changes on the binding affinity were observed to be context dependent. A systematic method to give high-quality estimates of such complex affinity landscapes is, thus, essential to the control of gene expression and the advance of synthetic biology.

**Results:**

Here, we propose a two-round prediction method that is based on support vector regression (SVR) with weighted degree (WD) kernels. In the first round, a WD kernel with shifts and mismatches is used with SVR to detect the importance of subsequences with different lengths at different positions. The subsequences identified as important in the first round are then fed into a second WD kernel to fit the experimentally measured affinities. To our knowledge, this is the first attempt to increase the accuracy of the affinity prediction by applying two rounds of string kernels and by identifying a small number of crucial k-mers. The proposed method was tested by predicting the binding affinity landscape of Gcn4p in *Saccharomyces cerevisiae *using datasets from HiTS-FLIP. Our method explicitly identified important subsequences and showed significant performance improvements when compared with other state-of-the-art methods. Based on the identified important subsequences, we discovered two surprisingly stable 10-mers and one sensitive 10-mer which were not reported before. Further test on four other TFs in *S. cerevisiae *demonstrated the generality of our method.

**Conclusion:**

We proposed in this paper a two-round method to quantitatively model the DNA binding affinity landscape. Since the ability to modify genetic parts to fine-tune gene expression rates is crucial to the design of biological systems, such a tool may play an important role in the success of synthetic biology going forward.

## Introduction

A major goal of synthetic biology is to manipulate existing organisms so as to construct new biological systems that possess desired functions [1-3]. The ability to adjust the expression of genes precisely is then necessary if the behavior of a synthetic biological system is to be fine-tuned for a given functional specification. Since the initiation of transcription is one of the most important steps in gene regulation [4], a quantitative understanding of interactions between transcription factors (TFs) and their DNA binding sites is key to predicting the dynamics of gene circuits. However, the mechanistic characterization of intricate TF-DNA interactions from first principles of biochemistry still remains elusive. Consequently, the use of phenomenological models to characterize the affinity of TF-DNA interactions is essential for the rational design of synthetic gene circuits.

There are several high-throughput methods available to perform high-resolution measurements of protein-DNA interactions. These include *protein-binding microarrays *(PBMs), which characterize *in vitro *binding specificities of TFs for relatively short DNA sequences [5], and *chromatin immunoprecipitation *(ChIP)-based methods, which, in a cell-type specific fashion, can map genome-wide binding locations of TFs, provided that the relevant antibodies are available [6]. *Mechanically induced trapping of molecular interactions *(MITOMI) was developed which is capable of detecting low affinity TF-DNA binding using a microfluidic device [7]. They further developed a second-generation of MITOMI that was capable of measuring thousands of interactions in parallel [8]. Recently, *high-throughput sequencing - fluorescent ligand interaction profiling *(HiTS-FLIP) has been developed [9]. This is a method based on a second-generation DNA sequencing technology, which allows for hundreds of millions of *in vitro *measurements of TF-DNA binding affinities and provides a more comprehensive picture of the binding affinity landscapes of TFs. Further, HiTS-FLIP permits measurements of longer sequences of DNA, making possible analysis of complex binding affinity landscapes of dimeric and oligomeric TFs.

Various statistical and computational models have been developed to characterize binding affinity [10,11]. The most commonly used one of these is the position weight matrix (PWM) [12,13]. The basic PWM model aligns the DNA binding sequences and calculates the weights of different nucleotides at different positions within the alignment. There are different variants of PWM. All PWM models, however, assume that mononucleotides at different positions contribute independently. Although such models provided relatively accurate predictions for short binding motifs [14], alternative models have been developed to encode short-range information through building much larger matrices for subsequences (*k*-mers) [5,15]. MDscan [16], for example, combined the word enumeration and position-specific weight matrix updating to iteratively approximate maximum a posteriori scoring function. Foat et al. proposed MatrixREDUCE [17], a statistical mechanics method that took high-throughput measurements of binding affinity as inputs and performed a least-squares fit to estimate the position specific affinity matrix that contained the relative energy contribution of each nucleotide at different positions. RankMotif++ [18] learned PWM motif models by maximum likelihood estimation of a probabilistic model for binding preferences.

Recent advances in high-throughput measurements of binding affinity and machine learning techniques have enabled the direct learning of the DNA-binding affinity landscape of TFs. A special case of the mismatch string kernel, di-mismatch kernel, has been proposed [19] that maps each binding sequence to a kernel space depending on similarity to all unique *k*-mers in the training data, for a fixed *k *and certain allowance of mismatches. Spectrum kernel has been applied to classify mammalian enhancers [20]. More recently, Annala et al. [21] proposed a linear model (HK*→*ME) that assumes the binding affinity to be the sum of the contributions of certain subsequences of the binding sequences. Their method was the best performer in the Dialogue for Reverse Engineering Assessment and Methods 5 (DREAM5) transcription factor/DNA motif recognition competition.

Despite the significant advances made in computational methods for modeling DNA-binding affinity landscapes, there are still bottlenecks that continue to hamper the progress. The major one is that although most existing methods assume that *k*-mers make an important contribution to the binding affinity, there is no systematic overview provided of the importance of all *k*-mers with different lengths at different positions. For example, the PWM model assumes mononucleotides to be independent [13], whereas the di-mismatch kernel reveals which of the *k*-mers are important for a specific length, but cannot determine at which positions the *k*-mers are important or whether those with shorter length also contribute to the affinity. Similarly, the HK*→*ME method used *k*-mers of specific lengths, i.e., all of the 4-6-mers as well as those 7- and 8-mers with the highest median intensity. This fact makes it difficult for the existing methods to well capture the important *k*-mers and their important positions.

In this paper, we propose a two-round support vector regression (SVR) method based on weighted degree (WD) kernels to overcome this bottleneck. In the first round, a WD kernel with shifts and mismatches is used with SVR to detect the importance of subsequences with different lengths at different positions. The identified subsequences are then fed into a second WD kernel to fit the experimentally measured affinities. Our method can systematically explore all the subsequences up to a certain length at all positions, and the results can easily be interpreted by users. We have applied this method to predict the binding affinity landscape of Gcn4p in *Saccharomyces cerevisiae *by using datasets from HiTS-FLIP. Through comparison with state-of-the-art predictors, we demonstrate that our method can provide significant improvements. We also demonstrate that our method can be straightforwardly used to visualize the importance of any *k*-mer at any position in binding sequences, thus to gain insights in the design of binding site sequences. Furthermore, we predict two high-affinity 10-mer motifs that are significantly more stable than the previously reported binding motifs. To evaluate the generalization power of our method, we further test it on the datasets from MITOMI2.0 [8] of four other TFs in *S. cerevisiae*, i.e., Cbf1p, Cin5p, Pho4p and Yap1p. Our method shows consistent improvements over state-of-the-art methods.

## Materials and methods

### Support vector regression

Support vector regression (SVR) is a supervised regression model [22]. Given training data {(*x*_1_*, y*_1_)*, . . . *, (*x_n_, y_n_*)*}*, where *x_i_*is a high-dimensional feature vector and y_i _is the corresponding real-valued variable, the goal is to learn a function *f *(*x*) that has at most ε deviation from *y*_i _for all the *x_i_*. In the case of linear SVR, we have

f(x)=〈w,x〉+b,

where 〈·,·〉 denotes the inner product and *w *denotes the coefficient vector trained in linear SVR. The optimization formulation of SVR is thus

$$\text{min}\;\;\;\;\frac{1}{2}||w|{|}^{2}+C\overset{n}{\underset{i=1}{\sum }}(\xi_{i}+\xi_{i}^{*}),$$

$$\text{subject to}\;\left\{\begin{array}{c} y_{i}-\langle w,x\rangle -b\leq \in +\xi_{i},\\ \langle w,x\rangle +b-y_{i}\leq \in +\xi_{i}^{*},\\ \xi_{i},\xi_{i}^{*}\geq 0, \end{array}\right)$$

where ξ_i _are $\xi_{i}^{*}$ are slack variables and *C >*0 is the trade-off constant. If the relationship between *x_i _*and *y_i _*is non-linear, SVR can perform non-linear regression by kernel tricks which implicitly map *x_i _*to higher-dimensional feature spaces, i.e., *f *(*x*) = 〈*w*, Φ(*x*)〉 + *b*, where Φ(*x*) is a kernel mapping representation.

### String kernels

String kernels are positive definite kernel functions defined on pairs of strings. The basic idea of string kernels is to map each string to a high-dimensional feature space and calculate the inner product of the two feature vectors. In other words, string kernels measure the similarity between pairs of strings. The more similar the two strings, **a **and **b**, the higher will be the value of the string kernel, *K*(**a**, **b**).

The two main types of string kernels are distribution-based kernels and *k *-mer-based kernels. Distribution-based kernels attempt to model uncertainties using random variables. Such kernels include the probability product kernel [23] and the spectral latent kernel [24]. Such kernels, however, require relatively long input sequences to capture the statistically meaningful distributions of subsequences.

In contrast to the distribution-based kernels, the *k *-mer-based string kernels essentially count all subsequences in the two sequences with lengths up to a pre-defined value and use these as features. A *k*-mer is a length-*k *subsequence in a sequence, **a**. There are several types of *k*-mer-based kernels each of which handles different assumptions. The spectrum kernel [25] maps each sequence into a feature space where each dimension counts the number of occurrences of a particular subsequence. The underlying assumption of the spectrum kernel is that the positions at which the subsequences occur are not important, rather the frequencies of their occurrences are the informative factor. Unlike the spectrum kernel, which is position independent, the weighted degree (WD) kernel [26] compares matches of subsequences at exact positions.

Specifically, let **a***_k _*(*i*) denote a *k*-mer starting at position *i *of **a**. A *d*-th degree WD kernel of two sequences, **a **and **b**, of length *L *is defined as

(1) $$k\left(a,b\right)=\overset{d}{\underset{k=1}{\sum }}\beta_{k}\overset{L-k+1}{\underset{i=1}{\sum }}I\left[a_{k}\left(i\right)=b_{k}\left(i\right)\right],$$

where *β_k _*are weights for different *k*-mers and I[⋅] is an indicator function such that it is 1 when the condition inside the bracket is true and 0 otherwise. From Eq. 1, the computational complexity of calculating WD kernel between two sequences **a **and **b **is *O*(*dL*).

To incorporate alternations in DNA sequences caused by the substitution, deletion, and insertion into the WD kernel, WD kernel with shifts and mismatches was proposed [27-29] as follows:

(2) $$\begin{array}{c} K\left(a,b\right)=\overset{d}{\underset{k=1}{\sum }}\overset{M}{\underset{m=0}{\sum }}\beta_{k,m}\overset{L-k+1}{\underset{i=1}{\sum }}\gamma i\overset{S\left(i\right)}{\underset{s=0,s+i<L}{\sum }}\omega_{s}\mu_{k,m,i,s,\text{a},\text{b}},\\ \mu_{k,m,i,s,\text{a},\text{b}},=I\left[{\text{a}}_{k}\left(i+s\right){=}_{m}{\text{b}}_{k}\left(i\right)\right]+I\left[{\text{a}}_{k}\left(i\right){=}_{m}{\text{b}}_{k}\left(i+s\right)\right], \end{array}$$

where *β_k,m _*are the weights for *k*-mers and *m *mismatches, *γ_i _*are the weights for different sequence positions, $\omega_{s}=\frac{1}{2\left(s+1\right)}$ are the weights assigned to shifts (in either direction) of extent *s*, and *S*(*i*) determines the shift range at position *i*. I[**a***_k _*(*i *+ *s*) =*_m _***b***_k _*(*i*)] equals to 1 if and only if **a***_k _*(*i *+ *s*) and **b***_k _*(*i*) differ by exactly *m *mismatches, and 0 otherwise. In this case, the computational complexity of calculating WD kernel between two sequences **a **and **b **is *O*(*dLs*). Thus, the total runtime to compute the kernel matrix for the entire training dataset is *O*(*n*^2^*dLs*).

### A two-round SVR with WD kernel method

**Figure 1:**
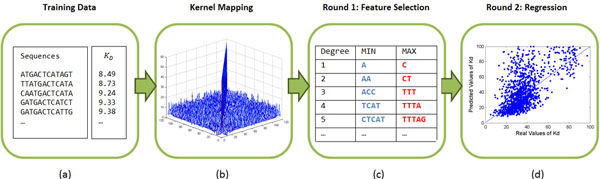
**The workflow of the proposed two-round support vector regression method with weighted degree kernels**. (a) The input training DNA binding site sequences with their corresponding *K_d _*values, demonstrating the general form of the inputs. (b) The weighted degree kernel matrix of the first round, calculated from Eq. 2. Each dimension lists the training binding sequences as shown in (a), and the corresponding entry value represents the similarity between the two sequences by the WD kernel. (c) Based on the kernel matrix in (b), we did the first round of support vector regression to select the top ten *k*-mers that contribute most to the high binding affinity (in blue) and the ten *k*-mers that contribute the most to the low binding affinity (in red). The local optimistic parameters were also selected from this step. (d) The regression of Round 2 to predict binding affinities by using the selected *k*-mers in a new WD kernel.

The workflow of the proposed two-round method is shown in Figure 1. The main idea is to use support vector regression with weighted degree kernels for both feature selection and regression.

In the first round, we map the input training sequences into a kernel space by the WD kernel with shift limit, *s*, and mismatch limit, *m*, according to Eq. 2. Here, all the *k*-mers up to length *d *are used. We then apply WD-kernel-based SVR to learn a model that maps DNA sequences and their *K_d _*values. The setting of *d, s *and *m *can be determined by the cross validation (CV) on the training set. From the learned SVR model, the top ten *k*-mers that contribute most to the high binding affinity (small *K_d _*values) and another ten that contribute the most to the low binding affinity (large *K_d _*values) for each *k *up to *d *are selected according to their expected decrease and increase of *f *(**x**), respectively, where *f *(*·*) is the learned regression model and **x **is an input sequence of length *L*. Here ten is the default parameter of our method and can be customized by users. Following [30], we quantify the importance of **x***_k _*(*i*), which represents a *k*-mer starting from position *i *of **x**, as *Q*(**x***_k _*(*i*)) = E[*f *(**x**)|**x***_k _*(*i*)] *− *E [*f *(**x**)]. In the second round, we learn another SVR model by encoding only the selected *k*-mers from Round 1 in the WD kernel. That is, when checking *k*-mers in the kernel function, we only count those matches that belong to the selected *k*-mers from the first round. In this round, since the important *k*-mers are supposed to be more conserved than other subsequences, we allow only shifts up to *s *but not mismatches, and, thus, follow Eq. 2. Again, the parameters *d *and *s *are set by cross-validation on the training set.

Once a test DNA binding sequence is given, it is mapped to a kernel space that is composed of the selected *k*-mers from Round 1. The learned SVR model in Round 2 is then applied to predict the *K_d _*value for this test binding sequence.

## Results

### Datasets

We first applied our method to predict the binding affinity landscape of Gcn4p in *Saccharomyces cerevisiae *by using datasets from HiTS-FLIP. Gcn4p is a master regulator that transcriptionally controls the expression of many genes including those for the amino acid biosynthesis pathway [31]. Gcn4p is a basic leucine zipper protein which interacts with DNA binding sites as a dimer [32], and is known to preferentially bind to several sequence motifs, including a 7-mer motif, TGACTCA [33,34]. On the basis of its important role in yeast in general control of amino acids [35], a quantitative characterization of the binding of Gcn4p to its promoter sites is crucial not only for elucidation of the regulatory mechanisms involved in such stress-response pathways, but also to allow design of a synthetic GCN4-induced response pathway in yeast.

The HiTS-FLIP datasets contain *K_d _*values of 83,252 DNA sequences of length 12bp. In TF-DNA interactions, *K_d _*represents the concentration of the TF at which the DNA region is occupied 50% of the time at equilibrium. The *K_d _*values in the HiTS-FLIP datasets range from 8 nM to 1000 nM, where a small *K_d _*represents high binding affinity and a large *K_d _*represents low binding affinity. Since the adjustment of binding-affinity for optimal TF-recognition sites is often important in the fine-tuning of the behavior of gene circuits, we focused on modeling the DNA-binding affinity landscape of Gcn4p with strong interactions. Here, DNA sequences with *K_d _*less than 100 nM were defined as optimal Gcn4p recognition sites. This threshold was chosen by looking up in the HiTS-FLIP datasets the *K_d _*value of a specific 9-mer, to which Gn4p was reported to bind relatively poorly *in vivo *and *in vitro *[34]. This resulted in 1,393 DNA sequences. This 12-mer dataset was randomly partitioned into 10 subsets to perform 10-fold CV. All the results of our method and other methods in this paper are based on the same 10-fold CV.

To evaluate the generalization power of our method, we further tested it on four other TF data sets of *S. cerevisiae*, i.e., Cbf1p, Cin5p, Pho4p and Yap1p. These four TFs are the ones measured by MITOMI2.0 [8]. Each data set contains the relative binding affinities for nucleotide sequences with 52bp in length, in which shorter binding sites are included. After removing the sequences with "nan" (not a number) and taking average relative affinities for the same sequences, each data set contains 1,456 52bp sequences, with their corresponding relative binding affinities.

### Performance measures

To evaluate the performance of regression methods, we measured the root mean square error (RMSE), root mean square relative error (RMSRE), Pearson product- moment correlation coefficient (Pearson Cor) and Spearman's rank correlation coefficient (Spearman Cor). These measures are defined as follows:

$$RMSE=\sqrt{\frac{1}{n}\overset{n}{\underset{i=1}{\sum }}{\left({y^{\prime }}_{i}-y_{i}\right)}^{2},}RMSRE=\sqrt{\frac{1}{n}\overset{n}{\underset{i=1}{\sum }}\left(\frac{{y^{\prime }}_{i}-y_{i}}{y_{i}}\right)2,}$$

$$Pearson\;Cor=\frac{\sum_{i=1}^{n}\left({y^{\prime }}_{i}-{{\bar{y}}^{\prime }}_{i}\right)\left(y_{i}-{\bar{y}}_{i}\right)}{\sqrt{\sum_{i=1}^{n}{\left({y^{\prime }}_{i}-{{\bar{y}}^{\prime }}_{i}\right)}^{2}}\sqrt{\sum_{i=1}^{n}{\left(y_{i}-{\bar{y}}_{i}\right)}^{2}}}$$

$$Spearman\;Cor=\frac{\sum_{i=1}^{n}\left({z^{\prime }}_{i}-{{\bar{z}}^{\prime }}_{i}\right)\left(z_{i}-{\bar{z}}_{i}\right)}{\sqrt{\sum_{i=1}^{n}{\left({z^{\prime }}_{i}-{{\bar{z}}^{\prime }}_{i}\right)}^{2}}\sqrt{\sum_{i=1}^{n}{\left(z_{i}-{\bar{z}}_{i}\right)}^{2}}}$$

where *y_i _*and $y_{i}^{\prime }$ are the real and predicted *K_d _*values, *z_i _*and ${z^{\prime }}_{i}$ are the real and predicted rank of *K_d _*values, for the *i*-th binding sequence respectively, and *n *is the number of binding sequences in the training or test sets.

### Results of 10-fold cross validation

**Figure 2:**
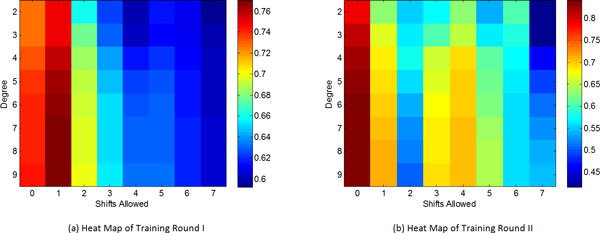
**Grid search of parameters on the training data of Rounds 1 and 2 of our method**. (a) Grid search of degree and shift for Round 1 in terms of the average *Pearson Cor *with mismatch 1. The parameter of mismatch can be searched in a similar manner, which is not shown here. (b) Grid search of degree and shift of for Round 2 in terms of *Pearson Cor*, with mismatch 0.

For selection of parameters (i.e., degree '*d*', shift '*s*' and mismatch '*m*'), a grid search for 2 *≤ d ≤ *9, 0 *≤ s ≤ *7, and 0 *≤ m ≤ min*(*d*, 3) on the training sets was conducted on both rounds of our method. When *d *increases above 7, there is no significant improvement in the performance, but the running time increases due to the much larger number of *k*-mers (Figure 2 and Table 1). Therefore, we chose the best parameter setting in terms of Pearson correlation coefficient for Round 1 as *d *= 7, *s *= 1, and *m *= 1 (Figure 2(a)). Important *k*-mers were thus identified and used as the WD kernel coding subsequences for Round 2. The same grid search was conducted on the training sets of the same 10-fold CV. The best shift and mismatch parameters are expected to be small for Round 2 because Round 1 already identified important positions of the selected *k*-mers. This is validated by the best parameter setting of *d *= 7, *s *= 0, and *m *= 0 (Figure 2(b)). By fixing *s *and *m *of the test sets to be the best ones identified on the training sets and grid searching for *d*, the performance on the test sets was consistent with that on the training sets. Both Round 1 and Round 2 had the best performance on the test sets when *d *= 7 (Table 1).

**Table 1:** Average prediction performance of the Rounds 1 and 2 of our method on test sets of the 10-fold CV.

Test Performance of Round 1: WD with s = 1 & m = 1				
d	**Runtime**	**RMSE**	**Pearson Cor**	**Spearman Cor**

2	572	20.06	0.74	0.46
3	1034	19.99	0.74	0.47
4	1448	19.87	0.75	0.48
5	1834	19.79	0.75	0.49
6	2221	19.77	0.75	0.49
**7**	**2430**	**19.76**	**0.75**	**0.50**
8	2908	19.75	0.75	0.50
9	3193	19.74	0.75	0.50

**Test Performance of Round 2: WD with s = 0 & m = 0**				

d	**Runtime**	**RMSE**	**Pearson Cor**	**Spearman Cor**

2	47	18.82	0.78	0.55
3	90	18.09	0.80	0.59
4	128	17.65	0.81	0.62
5	166	17.34	0.82	0.65
6	200	17.09	0.83	0.66
**7**	**235**	**16.89**	**0.84**	**0.68**
8	268	16.89	0.84	0.65
9	302	16.87	0.84	0.65

Round 1 uses all *k*-mers up to length *d*, with shift = 1 and mismatch = 1. Round 2 uses only selected *k*-mers from Round 1, with shift = 0 and mismatch = 0. 'Runtime' includes both training and testing, in seconds. The values for the parameters selected on training data are in bold.

Comparison of the relative performance of Rounds 1 and 2 reveals that Round 2 has significant improvements with respect to all the measures (Table 1). In particular, the RMSE, Pearson correlation and Spearman correlation of Round 2 are better than those of Round 1 by 15%, 12% and 36%, respectively. This is due to the fact that Round 1 encodes many irrelevant *k*-mers, whereas Round 2 encodes only the important *k*-mers identified in Round 1. The kernel mapping of Round 2 is thus far more accurate than that of Round 1. With *d *= 7, Round 1 encodes 21,844 *k*-mers to calculate the kernel function, whereas Round 2 encodes only 140 *k*-mers. This explains the significant improvement on the runtime of Round 2 over Round 1, although Round 2 requires inputs from Round 1.

**Figure 3:**
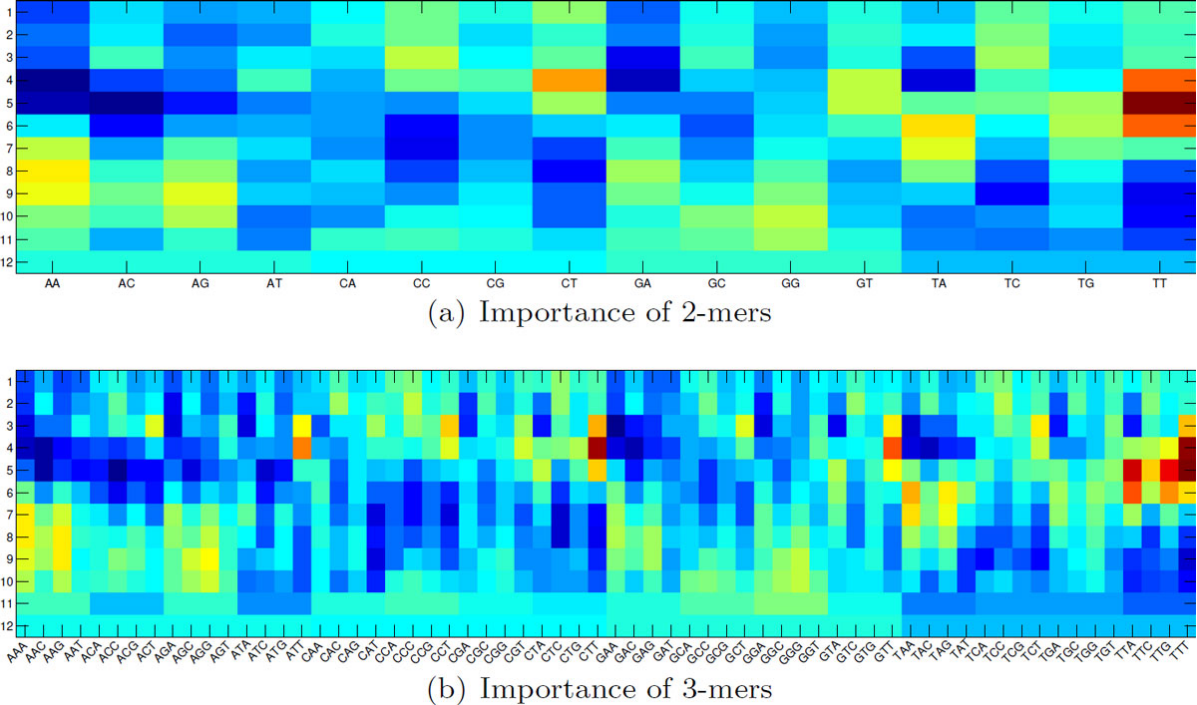
**The importance of all the (a) 2-mers and (b) 3-mers at different positions from Round 1. **The x-axis lays out all the 2-mers and 3-mers, respectively. The y-axis shows the positions within the 12-mer DNA binding sequence. The baseline color is yellow. Red color denotes the effect of leading to large *K_d _*values, whereas blue color denotes the effect of leading to small *K_d _*values.

The results from the 10-fold CV have demonstrated the effectiveness of the *k*-mer selection of Round 1. Figure 3 shows two illustrative examples of importance matrices for *k *= 2 and *k *= 3. The baseline color is yellow. The red color indicates that the *k*-mer at the corresponding starting position in the 12-mer binding sequence contributes to low binding affinity (a large *K_d _*value), whereas the blue color indicates a contribution to high binding affinity. For instance, TT at positions 4-6 tends to lead to a large *K_d _*value (Figure 3(a)). AA and AC, on the other hand, are preferred 2-mers at position 5. The effect of TT can be further decomposed into seven 3-mers as shown in Figure 3(b), that is, ATT, CTT, GTT, TTT, TTA, TTC, and TTG. Among them, CTT and TTT are those that contribute most to a large *K_d _*value if one of them appear at position 4 of the 12-mer Gcn4p-DNA binding sequence. It should be noticed that such importance matrices contain both uncertainty and diversity: uncertainty means that due to the effects of other contributing *k*-mers, a *k*-mer with a red color does not necessarily lead to a high *K_d _*value, and diversity means that multiple *k*-mers can contribute to *K_d _*values at the same position. Nevertheless, these importance matrices still provide an intuitive means for researchers to visualize and interpret results, and thus gain insights into the design of a binding sequence with a desired binding affinity.

### Comparison with state-of-the-art methods

Our method was further compared with state-of-the-art methods on the same datasets. HK*→*ME [21] was the best performer at the DREAM5 transcription factor/DNA motif recognition competition. According to Annala's method, HK*→*ME was set to use all the 4-6-mers as well as 2000 7-mers and 1000 8-mers with the lowest median *K_d _*values. We also compared with PWM which assumed the mononucleotide contributed independently to the binding affinity. The model *x *of PWM is solved from the function *A · × *= *K *where *A *is an *n × u *matrix, where *n *is the number of training sequences, and *u *= *L × z *where *L *is the length of each training sequence and *z *is the size of the dictionary. For the Gcn4p dataset, *L *is 12 and *z *is 4. Here *A*[*i, j*] is set to 1 if the *i*-th sequence contains the specific nucleotide at the specific position indicated by the index *j*, otherwise 0. The *x *is a *u*-dimensional column vector to be trained, each entry of which represents the weight for the corresponding nucleotide at the corresponding position. For the Gcn4p dataset, *K *is set to be *ln*(*K_d_*) because *ln*(*K_d_*) is proportional to the binding free energy, which is assumed to be additive.

Table 2 shows the comparison between our method and state-of-the-art methods on the same 10-fold CV. Our method significantly outperforms the other three methods. In particular, compared with HK*→*ME, our method scored 71% higher in the Pearson correlation and 51% higher in the Spearman correlation; compared with PWM, we have 50% higher in the Pearson correlation and 36% higher in the Spearman correlation. There are at least three reasons for these significant improvements. First, our method does not depend on prior knowledge of which *k*-mers are important, rather it systematically explores all *k*-mers up to length *d*. Secondly, our method selects the most important *k*-mers with different *k *values based on the expected importance of such subsequences at different positions, whereas PWM assumes 1-mers are important and HK*→*ME assumes certain *k*-mers are important without considering their positions and number of occurrence. Thirdly, the discriminative power of SVR ensures an accurate regression in the kernel space. We also implemented the SVR model with the WD kernel without shift or mismatch by *d *= 7 (as shown in Table 2) and found that it also significantly outperforms HK*→*ME and compares favorably to PWM. Our method, however, is clearly better than the SVR model on the basis that it allows shifts and mismatches, and conducts an additional round of *k*-mer selection.

**Table 2:** Comparison with state-of-the-art methods.

	PWM	HK*→*ME	SVR w. WD	Our Method
RMSE	20.2	25.4	22.5	**16.9**
RMSRE	46%	51%	58%	**44%**
Pearson Cor	0.56	0.49	0.70	**0.84**
Spearman Cor	0.50	0.45	0.50	**0.68**

"PWM" represents the position weight matrix model. "HK*→*ME" represents the linear model in [21]. "SVR w. WD" represents SVR with WD kernel without mismatch or shift. All values are the averages over the same 10-fold CV. The best values in each row are in bold.

### Discovery of a stable high-affinity 10-mer motif

**Figure 4:**
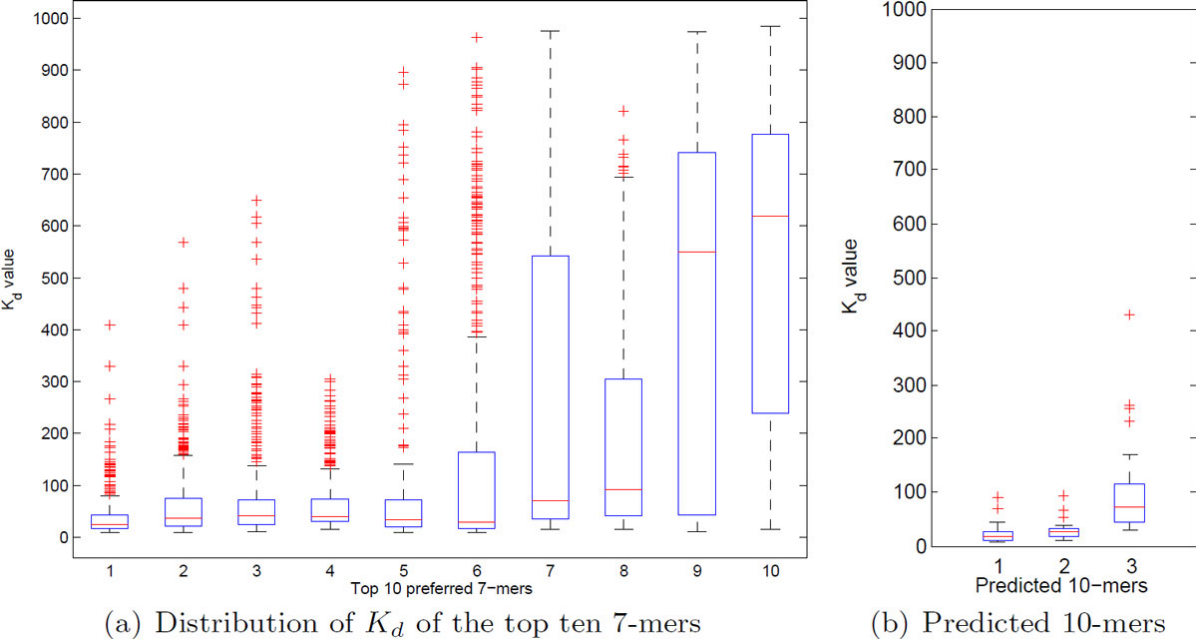
**Box plots of the 7-mers identified to be important for high-affinity 12-mers**. (a) The distribution of *K_d _*of the important 7-mers in the same order as in Table 3. (b) The distribution of *K_d _*of the three predicted 10-mers, including the stable 10-mers TATGACTCAT and TGTGACTCAT (the left two), and the sensitive 10-mer CATGACTAAT (the right one).

Since the two-round SVR model significantly increased the accuracy and efficiency of prediction to map DNA sequences to their *K_d _*values for the Gcn4p binding, we set out to characterize *k*-mers identified as being important through Round 1. In particular, we focused on the ten 7-mers that were selected to be important for high-affinity 12-mers. These ten 7-mers are listed in Table 3 and Figure 4. We noticed that these 7-mers appear relatively frequently throughout the 12-mer dataset, which contains DNA sequences with *K_d _*up to 1,000 nM. We measured statistics of the *K_d _*values of 12-mers composed of these important 7-mers, and found that six 7-mers, ATGACTC, TGACTCA, GTGACTC, TGAGTCA, TATGACT, and GACTCAT lead to a much lower dispersion of *K_d _*values than the other four important 7-mers (Figure 4). In other words, these six 7-mers are dominant factors that can, in most situations, stabilize the value of *K_d _*regardless of the context in which these 7-mers appear in nucleotide sequences.

**Table 3:** Statistics of the ten 7-mers that were identified to be important for high-affinity 12-mers through Round 1.

Rank	7-mer	Freq.	MIN	MAX	Average	Standard Deviation
1	**ATGACTC**	419	8.49	409.08	39.31	43.04
2	**TGACTCA**	990	8.49	567.81	56.66	54.61
3	**GTGACTC**	446	9.83	648.79	74.46	96.84
4	**TGAGTCA**	453	14.52	303.87	63.66	54.64
5	**TATGACT**	224	8.74	896.78	112.54	190.25
6	**GACTCAT**	392	8.49	963.28	167.26	254.46
7	ATGAGTC	504	15.60	975.18	276.01	292.93
8	TGACTAA	327	14.67	821.67	192.02	199.69
9	TACTCAC	847	9.65	975.05	437.92	336.43
10	GACTAAT	808	14.67	984.67	528.74	300.75

The seven columns list the rank of importance, nucleotide sequence, number of 12-mer sequences that contain this 7-mer, the minimum *K_d _*for all such 12-mers, the maximum *K_d _*for all such 12-mers, the mean *K_d _*value for these 12-mers, and the standard deviation of these 12-mers, respectively. The six 7-mers in bold are the ones with lower dispersions of *K_d _*values than the remainders.

Multiple sequence alignment of the six robust 7-mers reveals a high-affinity 10- mer motif, TRTGACTCAT. Interestingly, we found that, in the 12-mer dataset, the sequences composed of this 10-mer motif were guaranteed to have high binding affinity with the *K_d _*value being less than 100 nM. In addition, we found that this 10-mer motif leads to even lower variability in the *K_d _*values than the six 7-mers (Figure 4). Indeed, the mean and the standard deviation of all 12-mers composed of the two concrete sequences from this motif show high binding affinity with a low dispersion rate; TATGACTCAT has mean 23.89 nM and standard deviation nM, while TGTGACTCAT has mean 26.88 nM and standard deviation 18.25 nM. These results indicate that the high-affinity 10-mer motif we found is even more dominant factor that alone can stabilize *K_d _*at a low level. [9] confirmed that a palindromic 9-mer motif, ATGACTCAT, has a higher binding affinity than the consensus 7-mer motif, TGACTCA, as observed in previous studies [33,36]. While this 9-mer motif can indeed result in high binding affinity, it can also lead to much lower binding affinity than our 10-mer motif. For example, a 12-mer sequence, CATGACTCATAG, is observed to have *K_d _*value of 265.8 nM in the HiTS-FLIP dataset. Since the last 8 bases of the two motifs are the same (i.e., 5'-TGACTCAT- 3'), these observations indicate that our motif can further stabilize the *K_d _*values at a high affinity level by including an additional nucleotide in the left half-site. This is consistent with a previous experimental study in that, while Gcn4p binds to DNA sites as a dimer, the left half-site plays more important role than the right half-site in strong Gcn4p-DNA interactions [34].

In addition to the high-affinity 10-mer motif, we found a 10-mer sequence, CAT-GACTAAT, by performing multiple sequence alignment of 4 other 7-mers identified through Round 1 (i.e., ATGAGTC, TGACTAA, GACTCAC, and GACTAAT). Unlike the 10-mer motif that we found, however, this 10-mer is more context- dependent. While 12-mers composed of this 10-mer also gave relatively high mean binding affinity (*K_d _*= 97.88 nM), the range of the binding affinities increased significantly to include the *K_d _*values between 20.61 nM and 432.00 nM and to have the standard deviation of 90.65 nM (Figure 4). This shows that two nucleotide substitutions from the low-variance, high-affinity 10-mer motif can substantially alter the characteristics of the 10-mer. These observations indicate that the DNA binding affinity landscape of Gcn4p is very complex and that a strong interdependency is prevalent. This suggests that models based on additive, independent characteristics of binding free energy may not be able to quantitatively capture interactions of DNA and dimeric--and more generally oligomeric--TFs and that efficient models that consider interdependency of subsequences are key to understanding the DNA binding affinity landscape of such proteins and to fine-tuning of gene expression processes.

### Results on four other TFs in *S. cerevisiae*

To evaluate the generality of our method, we tested it on MITOMI2.0 datasets including four other TFs in *S. cerevisiae*, namely Cbf1p, Cin5p, Pho4p and Yap1p [8]. A same 5-fold CV was applied to evaluate each method. As shown in Tables 2 and 4, the Pearson correlation coefficient of different methods on these four TFs decrease significantly from that on Gcn4p. This makes sense because the input sequences in these four TFs are much longer than that in Gcn4p (52bp v.s. 12bp), which significantly increases the difficulty level of regression. Nevertheless, the outperformance of our method over the other methods is consistent with that on the Gcn4p dataset. This demonstrates the generality of our method and also suggests that our method can be applied to longer DNA sequences with high accuracy. The latter is essential to prediction of the binding affinity landscape of oligomeric TFs.

**Table 4:** Comparison with state-of-the-art methods on four other TFs in S. cerevisiae

	PWM	HK*→*ME	SVR w. WD	Our Method
Cbf1p	0.25	0.30	0.36	**0.63**
Cin5p	0.21	0.26	0.47	**0.62**
Pho4p	0.19	0.24	0.41	**0.61**
Yap1p	0.22	0.24	0.40	**0.58**

All values are *Pearson Cor *and averaged over the same 5-fold CV. The best values in each row are in bold.

## Conclusion

In this paper, we have proposed a novel two-round support vector regression method that is based on weighted degree kernels with shifts and mismatches, with the first round focusing on feature selection and the second round focusing on regression. The WD kernels have been used with support vector classification method and successfully applied to a number of biological sequence classification problems, including transcription start site prediction [37], splice site prediction [38], alternative splicing site prediction [28], trans-splicing site prediction [39], and translation initiation site prediction [40]. However, the power of combining the WD kernels with the support vector regression has not been well studied in bioinformatics. Further, to the best of our knowledge, two rounds of string kernels have not been applied to identify crucial k-mers and to avoid projecting the input sequences to overly high-dimensional kernel space.

We applied the proposed two-round method to model the mapping of DNA sequences to their binding affinity for the Gcn4p binding in yeast using high-resolution datasets measured by HiTS-FLIP. We showed that the quantitative prediction from our new method is significantly improved over existing methods. We further demonstrated that the identification of important subsequences would allow extraction of human-interpretable rules for the purpose of quantitative control of binding affinity. Two 10-mers were predicted by our method that were surprisingly stable but were not previously reported. Another 10-mer that just has two nucleotide changes from one of the stable ones was predicted that was comparatively sensitive. Additional tests on four other TFs validate the generalization power of the proposed method. Our program and sample data are freely available at http://sfb.kaust.edu.sa/Pages/Software.aspx.

## Competing interests

The authors declare that they have no competing interests.
